# Vascular complications and changes in body mass index in Japanese type 2 diabetic patients with abdominal obesity

**DOI:** 10.1186/1475-2840-12-88

**Published:** 2013-06-18

**Authors:** Hirofumi Nagao, Susumu Kashine, Hitoshi Nishizawa, Takuya Okada, Takekazu Kimura, Ayumu Hirata, Shiro Fukuda, Junji Kozawa, Norikazu Maeda, Tetsuhiro Kitamura, Tetsuyuki Yasuda, Kohei Okita, Toshiyuki Hibuse, Mamiko Tsugawa, Akihisa Imagawa, Tohru Funahashi, Iichiro Shimomura

**Affiliations:** 1 Department of Metabolic Medicine, Graduate School of Medicine, Osaka University, 2-2 B-5, Yamada-oka, Suita, Osaka 565-0871, Japan; 2 Suita Municipal Hospital, 2-13-20, Katayamacho, Suita, Osaka 564-0082, Japan; 3 Ikeda Municipal Hospital, 3-1-18, Jonan, Ikeda, Osaka 563-8510, Japan; 4 Department of Metabolism and Atherosclerosis, Graduate School of Medicine, Osaka University, 2-2-B, Yamada-oka, Suita, Osaka 565-0871, Japan

**Keywords:** Abdominal obesity, Type 2 diabetes, Waist circumference, Visceral fat accumulation, Body mass index, Cardiovascular disease

## Abstract

**Background:**

Although many Asian type 2 diabetic patients have been considered to be not obese and have low capacity of insulin secretion, the proportion of obese patients with visceral fat accumulation has increased in recent years. We found previously considerable number of Japanese non-obese subjects (body mass index (BMI) < 25 kg/m^2^) with visceral fat accumulation and multiple cardiovascular risk factors. The aim of the study was to investigate the difference in clinical features of type 2 diabetic patients with and without visceral fat accumulation, focusing on vascular complications and changes in BMI.

**Methods:**

We enrolled 88 Japanese hospitalized type 2 diabetic patients. Abdominal obesity represented waist circumference (WC) of ≥85 cm for males and ≥90 cm for females (corresponding to visceral fat area of 100 cm^2^). Subjects were divided into two groups; with or without abdominal obesity.

**Results:**

Hypertension, dyslipidemia and cardiovascular diseases were significantly more in the patients with abdominal obesity. The prevalence of cardiovascular disease in the non-obese patients (BMI < 25 kg/m^2^) with abdominal obesity were similar in obese patients (BMI ≥25 kg/m^2^). The mean BMI of the patients with abdominal obesity was < 25 kg/m^2^ at 20 years of age, but reached maximum to more than 30 kg/m^2^ in the course. Furthermore, substantial portion of the type 2 diabetic patients (52% in males and 43% in females) were not obese at 20 year-old (BMI < 25 kg/m^2^), but developed abdominal obesity by the time of admission.

**Conclusion:**

These results emphasize the need to control multiple risk factors and prevent atherosclerotic disease in patients with abdominal obesity. The significant weight gain after 20 years of age in patients with abdominal obesity stresses the importance of lifestyle modification in younger generation, to prevent potential development of type 2 diabetes and future atherosclerotic cardiovascular disease.

## Background

The prevalence of type 2 diabetes and obesity has increased worldwide, especially in Asia, in association with visceral fat accumulation [1-4]. Previous studies indicated that many Asian diabetic patients are not obese and have low capacity of insulin secretion [5-7]. However, recent evidence from some studies suggested that obesity and visceral fat accumulation were closely related to the incidence of type 2 diabetes [8-10]. Our group reported recently that obese type 2 diabetic patients secrete high insulin levels after oral glucose loading than non-obese patients [11].

Body mass index (BMI) is widely used to assess adiposity. Asians and Japanese could be easily affected with type 2 diabetes mellitus, including those with relatively low BMI compared to Caucasians [7]. Furthermore, even within the non-obese (BMI < 25 kg/m^2^), increase in BMI was reported to escalate the risk of type 2 diabetes mellitus in Japanese population [8]. We reported previously the identification of a considerable proportion of Japanese subjects with visceral fat accumulation (visceral fat area, VFA ≥100 cm^2^) whose BMI was less than 25 kg/m^2^, and that subjects with visceral fat accumulation without overall obesity (VFA ≥100 cm^2^ plus BMI < 25 kg/m^2^) had multiple cardiovascular risk factors [12]. There is ample evidence for the role of visceral fat accumulation in the development of multiple metabolic disorders including glucose intolerance, dyslipidemia, elevated blood pressure, and atherosclerotic cardiovascular diseases [13-20]. Collectively, these data suggest that assessment of visceral fat accumulation is useful for evaluation of high-risk group for atherosclerotic cardiovascular diseases, and also type 2 diabetes. However, to date, it has not been well characterized about the state of body weight in younger age of the current type 2 diabetic patients with visceral fat accumulation. Our group has reported previously that the mean visceral fat area increased with age after 20’s in general population [21]. It may be important to know whether current type 2 patients with visceral fat accumulation has been obese or not at 20 years of age, to prevent visceral fat accumulation.

Clinically, waist circumference (WC) is used as a tool for estimation of VFA. In Japanese population, VFA of 100 cm^2^ has been demonstrated to correspond approximately to WC of 85 cm in male and 90 cm in female [21,22]. Therefore, in Japanese guidelines for the metabolic syndrome, WC is used as an index of visceral fat accumulation for practical convenience [23].

The aim of this study is to investigate the difference in clinical features of type 2 diabetic patients with or without abdominal obesity (WC ≥85 cm in males, ≥90 cm in females), with a special focus on vascular complications and changes in BMI.

## Methods

### Subjects

The study subjects were 88 Japanese type 2 diabetic patients who had been hospitalized because of poor glycemic control and/or the staging of complications at three institutions: Department of Endocrinology and Metabolism in Osaka University Hospital during the period from January 2010 to December 2010 (consecutive 54 patients were enrolled), Ikeda Municipal Hospital during the period from July 2011 to December 2011 (consecutive 16 patients were enrolled), Suita Municipal Hospital during the period from August 2011 to March 2012 (consecutive 18 patients were enrolled). The Medical Ethics Committee of Osaka University approved the study. Each participant gave a written informed consent.

Type 2 diabetes was defined according to the World Health Organization (WHO) National diabetic group criteria of 2006 and/or treatment of diabetes. The following patients were excluded, (1) patients in whom WC was not measured, (2) patients who were younger than 40 years or older than 64 years on admission, (3) patients who were diagnosed with type 1 diabetes mellitus, (4) patients positive for anti-GAD antibody, (5) patients who did not attend the diabetes education course due to severity of their illness.

### Clinical examination

Body weight at 20 years of age and maximum body weight were retrieved through medical interview. The duration of diabetes and the number of hospitalizations were determined from the medical interview and medical records. Second-degree family history of diabetes was obtained from every patient. Height (cm), weight (kg) and WC at umbilical level (cm) were measured in the standing position on admission. Diabetic retinopathy was assessed by an ophthalmologist. The stages of diabetic nephropathy were classified as normoalbuminuria [urinary albumin-creatinine ratio (UACR): < 30 mg/g creatinine], microalbuminuria (UACR 30–299 mg/g creatinine) and overt proteinuria (UACR: ≥300 mg/g creatinine). Venous blood samples were collected after overnight fasting for measurement of high-density lipoprotein cholesterol, low-density lipoprotein cholesterol, triglyceride, insulin, C-peptide, creatinine and HbA1c (Japan Diabetes Society, JDS). The value for HbA1c (%) was estimated as National Glycohemoglobin Standardization Program (NGSP) equivalent value (%), calculated by the formula HbA1c (%) =1.02 × HbA1c(JDS)+0.25 [24]. The homeostasis model assessment of insulin resistance (HOMA-IR = fasting plasma glucose (mg/dL) × fasting immunoreactive insulin (μU/mL) / 405) was evaluated after achieving fair glycemic control (fasting plasma glucose <140 mg/dL [25]), but patients treated with insulin were excluded. The maximum intima-media thickness (IMT) of the carotid artery was measured in supine position by echography. Maximum carotid IMT was measured on both the right and left sides in the observation-possible areas of the common carotid artery, bulbus, and internal carotid artery, but not the external carotid artery.

### Definition of abdominal obesity, hypertension, dyslipidemia and cardiovascular disease

Abdominal obesity was assessed WC of ≥85 cm for males and ≥90 cm for females [21,22]. Hypertension was defined as systolic blood pressure of ≥140 mmHg and/or diastolic blood pressure of ≥90 mmHg. Dyslipidemia was defined as low-density lipoprotein cholesterol concentration of >140 mg/dL and/or triglyceride concentration of >150 mg/dL and/or high-density lipoprotein cholesterol concentration of < 40 mg/dL. If patients received anti-hypertensive and /or anti-lipidemic medications, they were considered positive for hypertension and /or dyslipidemia. Cardiovascular disease was defined as coronary artery disease (CAD) and/or cerebrovascular disease and/or peripheral arterial disease (PAD). CAD was defined as significant coronary stenosis(es) by coronary angiography or computed tomography**,** and/or positive ischemia by stress myocardial scintigraphy. Cerebrovascular disease was defined as history of stroke by a medical interview and/or old cerebral infarction by magnetic resonance imaging (MRI). PAD was defined as ankle-brachial index (ABI) ≤ 0.9 or performance of a revascularization procedure or amputation of lower extremity because of PAD [26,27].

### Statistical analysis

**Figure 1:**
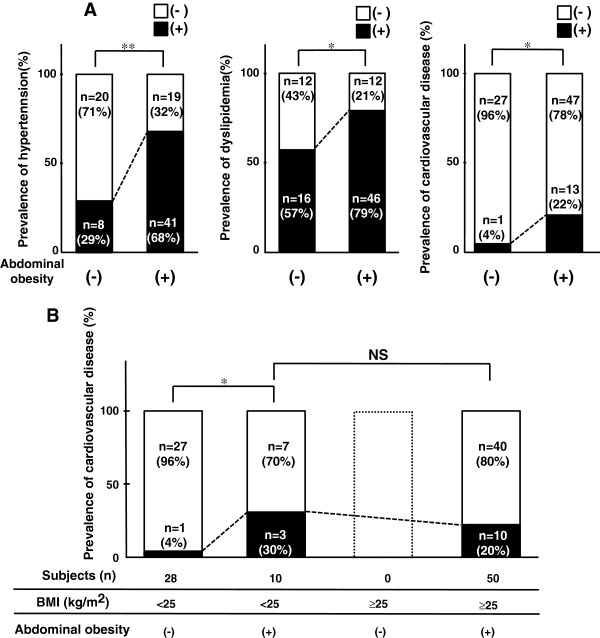
**Metabolic risk factors and cardiovascular disease in type 2 diabetic patients with abdominal obesity. **(**A**) Prevalence of hypertension, dyslipidemia and cardiovascular disease in type 2 diabetic patients with abdominal obesity. (**B**) Relationship between prevalence of cardiovascular disease and body fat distribution. Subjects were divided according to body mass index (BMI) and waist circumference (WC). Abdominal obesity represented WC of ≥85 cm in males and ≥90 cm in females. *P < 0.05, **P < 0.01.

**Figure 2:**
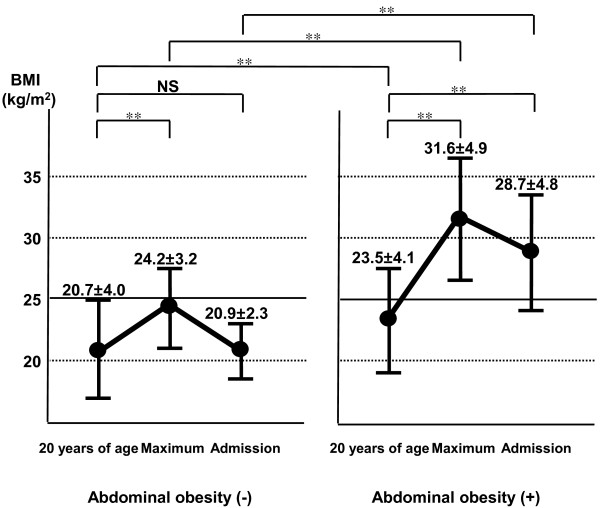
**Changes in BMI after 20 years of age in type 2 diabetic patients.** Data are mean ± SD. **P < 0.01.

All values were expressed as mean ± SD. In all cases, probability (P) values of < 0.05 were considered statistically significant. We performed unpaired *t*-test to determine the difference in various parameters between two groups (Table 1). Frequencies were compared between two groups by the Fisher’s exact test (Table 1, Figures 1). Changes in BMI in the same group were analyzed by paired *t*-test (Figure 2). Cochran-Armitage trend test was used to analyze the relationship between two groups of diabetic retinopathy and diabetic nephropathy (Table 1). All analyses were conducted by using the JMP version 9.0.2 for Windows (SAS Institute, Cary, NC).

**Table 1:** Baseline characteristics

	**Abdominal obesity**		**p value**
	**(−) group**	**(+) group**	
n	28	60	
Males/Females	13/15	38/22	0.167
Age, years	55.7 ± 6.7	54.8 ± 7.3	0.575
Body mass index, kg/m^2^	20.9 ± 2.3	28.7 ± 4.8	**<0.01**
Duration of diabetes mellitus, years	11.6 ± 10.3	9.1 ± 7.8	0.216
Number of hospitalizations	1.7 ± 0.9	2.3 ± 2.0	0.176
Family history of diabetes mellitus, %	61	53	0.646
Fasting plasma glucose, mg/dL	154 ± 56	153 ± 50	0.951
Hemoglobin A1c (NGSP), % (mmol/mol)	9.3 ± 2.2 (78)	9.1 ± 1.9 (76)	0.648
Fasting insulin, IU/L	4.6 ± 2.9	9.9 ± 8.4	**<0.01**
Fasting C-peptide, ng/mL	1.5 ± 0.9	2.0 ± 1.0	**<0.05**
HOMA-IR	1.5 ± 1.0 (n = 18)	3.4 ± 3.5 (n = 30)	**<0.05**
Maximum carotid IMT, mm	1.4 ± 0.7	1.6 ± 1.0	0.345
Diabetic retinopathy (NDR/SDR/PPDR/PDR)	16/5/0/7	41/7/3/8	0.250
Diabetic nephropathy (normo/micro/overt)	21/5/2	44/9/6	0.814
Discharge medications for diabetes mellitus			
Sulfonylurea, %	32	20	0.283
Biguanide, %	7	40	**<0.01**
Thiazolidinedione, %	14	12	0.738
Alpha-glucosidase inhibitor, %	18	15	0.760
Glinide, %	0	5	0.548
Dipeptidyl peptidase-4 inhibitor, %	18	27	0.431
Glucagon-like peptide-1 analog, %	0	8	0.173
Insulin, %	54	38	0.248

Data are mean ± SD, number of subjects or frequency [n (%)],

*NGSP* national glycohemoglobin standardization program, *HOMA-IR* homeostasis model assessment of insulin resistance, *IMT* intima-media thickness, *NDR* non-diabetic retinopathy, *SDR* simple diabetic retinopathy, *PPDR* preproliferative diabetic retinopathy, *PDR* proliferative diabetic retinopathy, *normo* normoalbuminuria, *micro* microalbuminuria, *overt* overt proteinuria.

## Results

### Characteristics of participants

We enrolled 88 Japanese patients in this study (51 men and 37 women; age, 55.1±7.1 years [mean ± SD]; range, 40–64 years, BMI 26.2±5.5 kg/m^2^, WC; males 91.4±9.8 cm, females 91.8±13.4 cm). We divided the patients into two groups by WC. Abdominal obesity was defined as WC ≥85 cm in males, WC ≥90 cm in females [19]. Patients with smaller WC formed the abdominal obesity (−) group. Table 1 summarizes the profiles of the 60 patients (68%) of the abdominal obesity (+) group and 28 patients (32%) of the abdominal obesity (−) group. There were no significant differences in age, duration of diabetes, family history of diabetes, HbA1c, fasting plasma glucose and maximum carotid IMT between the two groups. Furthermore, there were no significant differences in the percentage of patients with microangiopathies (diabetic retinopathy and diabetic nephropathy) between the two groups. On the other hand, BMI, fasting insulin, fasting C-peptide and HOMA-IR of the abdominal obesity (+) group were higher than those of the abdominal obesity (−) group.

### Clinical features of type 2 diabetic patients with abdominal obesity

Hypertension and dyslipidemia were significantly more common in the abdominal obesity (+) group compared with the abdominal obesity (−) group (68% vs 29% and 79% vs 57%, respectively, Figure 1A). Prevalence of cardiovascular diseases was higher in the abdominal obesity (+) group than the abdominal obesity (−) group (22% vs 4%, respectively, p < 0.05). Cardiovascular diseases in detail as follows; CAD 8cases (7 cases in abdominal obesity (+) group and 1cases in abdominal obesity (−) group; cerebrovascular disease 4 cases (abdominal obesity (+) group); and PAD 4 cases (abdominal obesity (+) group). Two patients with abdominal obesity suffered from two cardiovascular diseases, respectively (CAD + PAD and cerebrovascular disease + PAD).

Next, we investigated the relationship between prevalence of cardiovascular disease and body fat distribution. Subjects were divided according to their BMI and WC (Figure 1B). Among 88 patients, 50 patients (57%) were obese (BMI ≥25 kg/m^2^) with abdominal obesity. Among the non-obese (BMI < 25 kg/m^2^) patients, abdominal obesity was common (10/38: 26%) and the prevalence of cardiovascular disease was significantly higher, compared with the abdominal obesity (−) group. The prevalence of cardiovascular disease in non-obese (BMI < 25 kg/m^2^) patients with abdominal obesity was similar to that in obese (BMI ≥25 kg/m^2^) patients.

### Changes in BMI after 20 years of age in type 2 diabetics with abdominal obesity

We compared body weight at 20 years of age, at maximum and at admission to clarify changes in BMI in diabetic patients with and without abdominal obesity (Figure 2). The body weight at 20 years of age and the maximum weight were obtained at the medical interview. The mean BMI of the abdominal obesity (−) group was < 25 kg/m^2^ at 20 years of age, at maximum and at admission. On the other hand, the mean BMI of the abdominal obesity (+) group was 23.5 kg/m^2^ at 20 years of age (non-obese), but reached maximum to more than 30 kg/m^2^ in the course. The BMI at 20 years of age was significantly higher in the abdominal obesity (+) group than in the abdominal obesity (−) group (23.5 kg/m^2^ vs 20.7 kg/m^2^, respectively).

**Figure 3:**
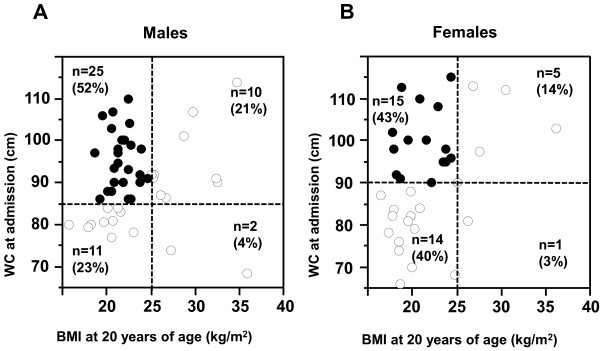
Relationship between WC at admission and BMI at 20 years of age in (A) males (B) females.

Finally, we examined the relationship between WC at admission and BMI at 20 years of age in all subjects with type 2 diabetes (Figure 3). 52% (n = 25) in males and 43% (n = 15) in females of the type 2 diabetic patients were not obese at 20 year-old (BMI < 25 kg/m^2^), but developed abdominal obesity by the time of admission.

## Discussion

The purpose of this cross-sectional study was to clarify the clinical features of type 2 diabetic patients with abdominal obesity. In the present study, we demonstrated the following differences between the abdominal obesity (+) group and the abdominal obesity (−) group; 1) Hypertension and dyslipidemia were significantly more prevalent in type 2 diabetic patients with abdominal obesity. 2) Cardiovascular diseases were significantly more in patients with abdominal obesity. 3) There were no significant differences in the prevalence of diabetic microangiopathies (diabetic retinopathy and diabetic nephropathy) between the two groups. 4) In the abdominal obesity (+) group, the mean BMI was < 25 kg/m^2^ at 20 years of age (non-obese), but reached maximum to more than 30 kg/m^2^ in the course. 5) Substantial portion of the type 2 diabetic patients (52% in males and 43% in females) were not obese at 20 year-old (BMI < 25 kg/m^2^), but developed abdominal obesity by the time of admission.

### Abdominal obesity and atherosclerotic cardiovascular disease

Visceral fat accumulation is accompanied by lifestyle changes, such as disturbance of eating habits, physical inactivity and sleep disorders. The hypertrophied adipocytes in intra-abdominal visceral fat exhibit hyperlipolytic activity, which is resistant to the antilipolytic effect of insulin [28,29]. The resulting hyper-free-fatty-acidemia and hyperglycerolemia in the portal vein increase the production of triacylglycerol-rich lipoproteins [30-33] and glucose in the liver [34,35], leading to dyslipidemia, insulin resistance and diabetes. Adipose tissue is not only an energy-storage organ, but it produces and secretes a variety of biologically active molecules known as adipocytokines, such as adiponectin, PAI-1 and TNF-α [15,16,36,37]. Dysregulation of adipocytokines caused by visceral fat accumulation is regarded as one of the major pathophysiological mechanisms of atherosclerosis associated with the metabolic syndrome. Our group has demonstrated that some of anti-diabetic, anti-hypertensive, and anti-dyslidemic agents had potential to target obesity and hypoadiponectinemia [38-42].

In Japanese guidelines for the metabolic syndrome, WC is used as an index of visceral fat accumulation for practical convenience [23]. Metabolic syndrome is defined as those having visceral fat accumulation expressed by increased WC (WC ≥85 cm in males, ≥90 cm in females) and 2 or more risk factors [23]. In the present study, abdominal obesity was defined as WC ≥85 cm in males and WC ≥90 cm in females. Thus, visceral fat accumulation is suspected to be present in type 2 diabetic patients with abdominal obesity. Hypertension, dyslipidemia and cardiovascular disease were significantly more in type 2 diabetic patients with abdominal obesity than patients without such obesity. To prevent atherosclerotic cardiovascular disease (diabetic macroangiopathy), it is necessary to search thoroughly for atherosclerotic cardiovascular disease in type 2 diabetes patients with abdominal obesity. On the other hand, there were no significant differences in the prevalence of diabetic microangiopathies (diabetic retinopathy and diabetic nephropathy) between the two groups, suggesting that it is necessary to rule out the presence of microangiopathy in all patients with type 2 diabetes, regardless of abdominal obesity. Recent reports have demonstrated that the development and progression of diabetic microangiopathy is influenced by glycemic control and duration of diabetes [43-46], suggesting that it is necessary to search thoroughly for diabetic microangiopathy in the patients with poor glycemic control and/or long duration of diabetes.

In our study of Japanese male urban workers [12], visceral fat accumulation (VFA ≥100 cm^2^) was observed in a relatively large proportion of patients with BMI < 25 kg/m^2^ (non-obese) (26.8%). In the same study, we have demonstrated the presence of multiple risk factors in such subjects [12]. From the viewpoint of abdominal obesity (estimation of VFA from WC), we divided the participants in the present study (type 2 diabetics) into four groups according to BMI and presence of abdominal obesity (Figure 1B), and demonstrated the relationship of adiposity with the prevalence of cardiovascular disease. Abdominal obesity was recognized in all patients with BMI ≥25 kg/m^2^, and observed in relatively large numbers (n = 10: 26%) of non-obese patients (BMI < 25 kg/m^2^). The prevalence of cardiovascular disease in such patients with abdominal obesity (+) plus BMI < 25 kg/m^2^ was similar to that in obese patients (BMI ≥25 kg/m^2^), and significantly higher than in patients without abdominal obesity and BMI < 25 kg/m^2^. These results suggest that WC, which reflects visceral fat accumulation, is more useful for evaluation of the risk of cardiovascular disease in type 2 diabetic patients, compared with BMI. The present study also suggests that measurement of WC should be more important in patients with BMI < 25 kg/m^2^ (non-obese) for the prevention of clustering of multiple risk factors and cardiovascular disease, because all patients with BMI ≥25 kg/m^2^ had abdominal obesity.

### Weight gain after 20 years of age in type 2 diabetic patients with abdominal obesity

We analyzed body weight at 20 years of age and at maximum obtained through medical interview, and determined the changes in BMI in type 2 diabetic patients with abdominal obesity. Interestingly, the mean BMI was < 25 kg/m^2^ at 20 years of age (non-obese), but reached maximum to more than 30 kg/m^2^ in the course (Figure 2). Substantial portion of the type 2 diabetic patients (52% in males and 43% in females) were not obese at 20 year-old (BMI < 25 kg/m^2^), but developed abdominal obesity by the time of admission (Figure 3), suggesting that environmental factors, such as disturbance of eating habits, physical inactivity and sleep disorders, affected visceral fat accumulation after 20 years of age in these patients.

Recent researches reported that subjects with abdominal obesity were more likely to develop diabetes for five years and had higher mortality within after 13 years than those without abdominal obesity [47,48]. Considered together, our results suggest that intervention to individuals with abdominal obesity such as health education and health guidance after health checkup should be important in reducing the risk of future diabetes and cardiovascular disease after 20 years of age. Our results also showed that the mean BMI at 20 years of age was already higher in the abdominal obesity (+) group than in the abdominal obesity (−) group, suggesting that early intervention such as health education and diet control during childhood and adolescence at school and home is important to reduce the risk of future cardiovascular diseases.

The present study has several limitations. The study was not prospective in design, and included a relatively small population limited to Japanese, who have different physique from Caucasians.

## Conclusions

The present study demonstrated higher prevalence of hypertension, dyslipidemia and cardiovascular disease among type 2 diabetic patients with abdominal obesity and probable visceral fat accumulation compared to those without abdominal obesity. Furthermore, significant weight gain was observed in type 2 diabetic patients with abdominal obesity after 20 years of age. These results suggest that education on lifestyle modification in younger generation, to prevent visceral fat accumulation is important for the prevention of type 2 diabetes and future atherosclerotic cardiovascular disease**.**

## Consent

Written informed consent was obtained from the patient for the publication of this report and any accompanying images.

## Abbreviations

ABI: Ankle-brachial index; BMI: Body mass index; CAD: Coronary artery disease; HOMA-IR: The homeostasis model assessment of insulin resistance; IMT: Intima-media thickness; JDS: Japan Diabetes Society; MRI: Magnetic resonance imaging; NGSP: National Glycohemoglobin Standardization Program; PAD: Peripheral arterial disease; UACR: The urinary albumin-creatinine ratio; VFA: Visceral fat area; WC: Waist circumference; WHO: World Health Organization.

## Competing interests

The authors declare no conflict of interest.

## Authors’ contributions

HN and SK acquired and analyzed data, and wrote the manuscript. HN conceived study, analyzed data, and wrote the manuscript. TO, TK, AH, SF, JK, NM, TK, TY, KO, TH, MT, and AI acquired and researched data. TF and IS reviewed the manuscript. All authors read and approved the final manuscript.

## References

[B1] World Health Organization Obesity and overweight; WHO Fact Sheet No311 2013 **http://www.who.int/mediacentre/factsheets/fs311/en/index.html**23785677

[B2] Shaw JE Sicree RA Zimmet PZ Global estimates of the prevalence of diabetes for 2010 and 2030 Diabetes Res Clin Pract 2010 87 4 14 10.1016/j.diabres.2009.10.007 19896746

[B3] Yoon KH Lee JH Kim JW Cho JH Choi YH Ko SH Zimmet P Son HY Epidemic obesity and type 2 diabetes in Asia Lancet 2006 368 1681 1688 10.1016/S0140-6736(06)69703-1 17098087

[B4] Chan JC Malik V Jia W Kadowaki T Yajnik CS Yoon KH Hu FB Diabetes in Asia. epidemiology, risk factors, and pathophysiology JAMA 2009 301 2129 2140 10.1001/jama.2009.726 19470990

[B5] Fujimoto WY Overview of non-insulin-dependent diabetes mellitus (NIDDM) in different population groups Diabet Med 1996 13 S7 S10 8894472

[B6] Fujimoto WY Akanuma Y Kanazawa Y Mashiko S Leonetti D Wahl P Plasma insulin levels in Japanese and Japanese-American men with type 2 diabetes may be related to the occurrence of cardiovascular disease Diabetes Res Clin Pract 1989 6 121 127 10.1016/0168-8227(89)90116-2 2647443

[B7] Huxley R James WP Barzi F Patel JV Lear SA Suriyawongpaisal P Janus E Caterson I Zimmet P Prabhakaran D Reddy S Woodward M Obesity in Asia Collaboration. Ethnic comparisons of the cross-sectional relationships between measures of body size with diabetes and hypertension Obes Rev 2008 9 53 61 10.1111/j.1467-789X.2007.00439.x 18307700

[B8] Nagaya T Yoshida H Takahashi H Kawai M Increases in body mass index, even within non-obese levels, raise the risk for Type 2 diabetes mellitus: a follow-up study in a Japanese population Diabet Med 2005 22 1107 1111 10.1111/j.1464-5491.2005.01602.x 16026381

[B9] Oh SW Shin SA Yun YH Yoo T Huh BY Cut-off point of BMI and obesity-related comorbidities and mortality in middle-aged Koreans Obes Res 2004 12 2031 2040 10.1038/oby.2004.254 15687405

[B10] Neeland IJ Ayers CR Rohatgi AK Turer AT Berry JD Das SR Vega GL Khera A McGuire DK Grundy SM de Lemos JA Associations of visceral and abdominal subcutaneous adipose tissue with markers of cardiac and metabolic risk in obese adults Obesity (Silver Spring) 2012 Epub ahead of print 10.1002/oby.20135 PMC3751977 23687099

[B11] Iwahashi H Okauchi Y Ryo M Noguchi M Morita S Kishida K Okita K Ohira T Funahashi T Nakamura T Imagawa A Shimomura I Insulin-secretion capacity in normal glucose tolerance, impaired glucose tolerance, and diabetes in obese and non-obese Japanese patients J Diabetes Invest 2012 3 271 275 10.1111/j.2040-1124.2011.00180.x PMC4014949 24843576

[B12] Okauchi Y Nishizawa H Funahashi T Ogawa T Noguchi M Ryo M Kihara S Iwahashi H Yamagata K Nakamura T Shimomura I Matsuzawa Y Reduction of visceral fat is associated with decrease in the number of metabolic risk factors in Japanese men Diabetes Care 2007 30 2392 2394 10.2337/dc07-0218 17563343

[B13] Kissebah AH Vydelingum N Murray R Evans DJ Hartz AJ Kalkhoff RK Adams PW Relation of body fat distribution to metabolic complications of obesity J Clin Endocrinol Metab 1982 54 254 260 10.1210/jcem-54-2-254 7033275

[B14] Nakamura T Tokunaga K Shimomura I Nakamura T Tokunaga K Shimomura I Nishida M Yoshida S Kotani K Islam AH Keno Y Kobatake T Nagai Y Fujioka S Tarui S Matsuzawa Y Contribution of visceral fat accumulation to the development of coronary artery disease in non-obese men Atherosclerosis 1994 107 239 246 10.1016/0021-9150(94)90025-6 7980698

[B15] Shimomura I Funahashi T Takahashi M Maeda K Kotani K Nakamura T Yamashita S Miura M Fukuda Y Takemura K Tokunaga K Matsuzawa Y Enhanced expression of PAI-1 in visceral fat: possible contributor to vascular disease in obesity Nat Med 1996 2 800 803 10.1038/nm0796-800 8673927

[B16] Hotmisligli GS Arner P Caro JF Atkinson RL Spiegelman BM Increased adipose tissue expression of tumor necrosis factor-α in human obesity and insulin resistance J Clin Invest 1995 95 2409 2415 10.1172/JCI117936 7738205 PMC295872

[B17] Lindsay RS Funahashi T Hanson RL Matsuzawa Y Tanaka S Tataranni PA Knowler WC Krakoff J Adiponectin and development of type 2 diabetes in the Pima Indian population Lancet 2002 360 57 58 10.1016/S0140-6736(02)09335-2 12114044

[B18] Ryo M Nakamura T Kihara S Kumada M Shibazaki S Takahashi M Nagai M Matsuzawa Y Funahashi T Adiponectin as a biomarker of the metabolic syndrome Circ J 2004 68 975 981 10.1253/circj.68.975 15502375

[B19] Mallamaci F Zoccali C Cuzzola F Tripepi G Cutrupi S Parlongo S Tanaka S Ouchi N Kihara S Funahashi T Matsuzawa Y Adiponectin in essential hypertension J Nephrol 2002 15 507 511 12455716

[B20] Mohammadreza B Farzad H Davoud K Fereidoun Prof AF Prognostic significance of the complex “Visceral Adiposity Index” vs. simple anthropometric measures: Tehran lipid and glucose study Cardiovasc Diabetol 2012 11 20 22394430 10.1186/1475-2840-11-20 PMC3376032

[B21] Hiuge-Shimizu A Kishida K Funahashi T Ishizaka Y Oka R Okada M Suzuki S Takaya N Nakagawa T Fukui T Fukuda H Watanabe N Yoshizumi T Nakamura T Matsuzawa Y Yamakado M Shimomura I Absolute value of visceral fat area measured on computed tomography scans and obesity-related cardiovascular risk factors in large-scale Japanese general population (the VACATION-J study) Ann Med 2012 44 82 92 10.3109/07853890.2010.526138 20964583

[B22] New criteria for ‘obesity disease’ in Japan. Examination Committee of Criteria for ‘Obesity Disease’ in Japan; Japan Society for the Study of Obesity New criteria for ‘obesity disease’ in Japan. Examination Committee of Criteria for ‘Obesity Disease’ in Japan Circ J 2002 66 987 992 10.1253/circj.66.987 12419927

[B23] Teramoto T Sasaki J Ueshima H Egusa G Kinoshita M Shimamoto K Daida H Biro S Hirobe K Funahashi T Yokote K Yokode M Metabolic syndrome J Atheroscler Thromb 2008 15 1 5 10.5551/jat.E580 18319538

[B24] Kashiwagi A Kasuga M Araki E Oka Y Hanafusa T Ito H Tominaga M Oikawa S Noda M Kawamura T Sanke T Namba M Hashiramoto M Sasahara T Nishio Y Kuwa K Ueki K Takei I Umemoto M Murakami M Yamakado M Yatomi Y Ohashi H International clinical harmonization of glycated hemoglobin in Japan : From Japan Diabetes Society to National Glycohemoglobin Standardization Program values J Diabetes Invest 2012 3 39 40 10.1111/j.2040-1124.2012.00207.x PMC4014931 24843544

[B25] Yki-Järvinen H Esko N Eero H Marja-Riitta T Clinical benefits and mechanisms of a sustained response to intermittent insulin therapy in type 2 diabetic patients with secondary drug failure Am J Med 1988 84 185 92 10.1016/0002-9343(88)90412-3 3044067

[B26] Hiatt WR Medical Treatment of Peripheral Arterial Disease and Claudication N Engl J Med 2001 344 1608 1621 10.1056/NEJM200105243442108 11372014

[B27] Mohler ER 3rd Peripheral arterial disease: identification and implications Arch Intern Med 2003 163 2306 2314 10.1001/archinte.163.19.2306 14581250

[B28] Smith U Hammersten J Björntorp P Kral JG Regional differences and effect of weight reduction on human fat cell metabolism Eur J Clin Invest 1979 9 327 332 10.1111/j.1365-2362.1979.tb00892.x 118025

[B29] Mittelman SD Van Citters GW Kirkman EL Bergman RN Extreme insulin resistance of the central adipose depot in vivo Diabetes 2002 51 755 761 10.2337/diabetes.51.3.755 11872676

[B30] Londos C Brasaemle DL Schultz CJ Adler-Wailes DC Levin DM Kimmel AR Rondinone CM On the control of lipolysis in adipocytes Ann N Y Acad Sci 1999 892 155 168 10.1111/j.1749-6632.1999.tb07794.x 10842661

[B31] Kuriyama H Yamashita S Shimomura I Funahashi T Ishigami M Aragane K Miyaoka K Nakamura T Takemura K Man Z Toide K Nakayama N Fukuda Y Lin MC Wetterau JR Matsuzawa Y Enhanced expression of hepatic acyl-coenzyme A synthetase and microsomal triglyceride transfer protein messenger RNAs in the obese and hypertriglyceridemic rat with visceral fat accumulation Hepatology 1998 27 557 562 10.1002/hep.510270233 9462657

[B32] White DA Bennett AJ Billett MA Salter AM The assembly of triacylglycerol-rich lipoproteins: an essential role for the microsomal triacylglycerol transfer protein Br J Nutr 1998 80 219 229 9875061

[B33] Adeli K Taghibiglou C Van Iderstine SC Lewis GF Mechanisms of hepatic very low-density lipoprotein overproduction in insulin resistance Trends Cardiovasc Med 2001 11 170 176 10.1016/S1050-1738(01)00084-6 11597827

[B34] Kuriyama H Shimomura I Kishida K Kondo H Furuyama N Nishizawa H Maeda N Matsuda M Nagaretani H Kihara S Nakamura T Tochino Y Funahashi T Matsuzawa Y Coordinated regulation of fat-specific and liver-specific glycerol channels, aquaporin adipose and aquaporin 9 Diabetes 2002 51 2915 2921 10.2337/diabetes.51.10.2915 12351427

[B35] Maeda N Funahashi T Shimomura I Metabolic impact of adipose and hepatic glycerol channels aquaporin 7 and aquaporin 9 Nat Clin Pract Endocrinol Metab 2008 4 627 34 18852723 10.1038/ncpendmet0980

[B36] Spiegelman BM Flier JS Obesity and the regulation of energy balance Cell 2001 23 531 543 11239410 10.1016/s0092-8674(01)00240-9

[B37] Matsuzawa Y Funahashi T Nakamura T The concept of metabolic syndrome: contribution of visceral fat accumulation and its molecular mechanism J Atheroscler Thromb 2011 18 629 639 10.5551/jat.7922 21737960

[B38] Maeda N Takahashi M Funahashi T Kihara S Nishizawa H Kishida K Nagaretani H Matsuda M Komuro R Ouchi N Kuriyama H Hotta K Nakamura T Shimomura I Matsuzawa Y PPARgamma ligands increase expression and plasma concentrations of adiponectin, an adipose-derived protein Diabetes 2001 50 2094 2099 10.2337/diabetes.50.9.2094 11522676

[B39] Kurata A Nishizawa H Kihara S Maeda N Sonoda M Okada T Ohashi K Hibuse T Fujita K Yasui A Hiuge A Kumada M Kuriyama H Shimomura I Funahashi T Blockade of Angiotensin II type-1 receptor reduces oxidative stress in adipose tissue and ameliorates adipocytokine dysregulation Kidney Int 2006 70 1717 1724 10.1038/sj.ki.5001810 16985520

[B40] Hiuge A Tenenbaum A Maeda N Benderly M Kumada M Fisman EZ Tanne D Matas Z Hibuse T Fujita K Nishizawa H Adler Y Motro M Kihara S Shimomura I Behar S Funahashi T Effects of peroxisome proliferator-activated receptor ligands, bezafibrate and fenofibrate, on adiponectin level Arterioscler Thromb Vasc Biol 2007 27 635 641 10.1161/01.ATV.0000256469.06782.d5 17194889

[B41] Inoue K Maeda N Kashine S Fujishima Y Kozawa J Hiuge-Shimizu A Okita K Imagawa A Funahashi T Shimomura I Short-term effects of liraglutide on visceral fat adiposity, appetite, and food preference: a pilot study of obese Japanese patients with type 2 diabetes Cardiovasc Diabetol 2011 10 109 10.1186/1475-2840-10-109 22132774 PMC3260096

[B42] Fujishima Y Maeda N Inoue K Kashine S Nishizawa H Hirata A Kozawa J Yasuda T Okita K Imagawa A Funahashi T Shimomura I Efficacy of liraglutide, a glucagon-like peptide-1 (GLP-1) analogue, on body weight, eating behavior, and glycemic control, in Japanese obese type 2 diabetes Cardiovasc Diabetol 2012 11 107 10.1186/1475-2840-11-107 22973968 PMC3459720

[B43] Yoshida Y Hagura R Hara Y Sugasawa G Akanuma Y Risk factors for the development of diabetic retinopathy in Japanese type 2 diabetic patients Diabetes Res Clin Pract 2001 51 195 203 10.1016/S0168-8227(00)00212-6 11269892

[B44] Stratton IM Kohner EM Aldington SJ Turner RC Holman RR Manley SE Matthews DR UKPDS 50 risk factors for incidence and progression of retinopathy in Type II diabetes over 6 years from diagnosis Diabetologia 2001 44 156 163 10.1007/s001250051594 11270671

[B45] The Diabetes Control and Complications Trial Research Group The effect of intensive treatment of diabetes on the development and progression of long-term complications in insulin-dependent diabetes mellitus N Engl J Med 1993 329 977 986 8366922 10.1056/NEJM199309303291401

[B46] UK Prospective Diabetes Study (UKPDS) Group Intensive blood-glucose control with sulphonylureas or insulin compared with conventional treatment and risk of complications in patients with type 2 diabetes (UKPDS 33) Lancet 1998 352 837 853 9742976

[B47] Lee IT Chiu YF Hwu CM He CT Chiang FT Lin YC Assimes T Curb JD Sheu WH Central obesity is important but not essential component of the metabolic syndrome for predicting diabetes mellitus in a hypertensive family-based cohort. Results from the Stanford Asia-pacific program for hypertension and insulin resistance (SAPPHIRe) Taiwan follow-up study Cardiovasc Diabetol 2012 11 43 10.1186/1475-2840-11-43 22537054 PMC3476431

[B48] Nooyens AC van Duijnhoven FJ Verschuren WM Boer JM van der A DL All-cause mortality risk of metabolically healthy abdominal obese individuals: The EPIC-MORGEN study Obesity (Silver Spring) 2013 Epub ahead of print 10.1002/oby.20480 23595997

